# T119. CAN SOME YOUNG PEOPLE RECOVER FROM FIRST-EPISODE PSYCHOSIS WITH INTEGRATED PSYCHOSOCIAL TREATMENT WITHOUT ANTIPSYCHOTIC MEDICATIONS? AN RCT TO ASSESS RISKS, BENEFITS, AND RANGE OF OUTCOMES

**DOI:** 10.1093/schbul/sby016.395

**Published:** 2018-04-01

**Authors:** Patrick McGorry, Shona Francey, Barnaby Nelson, Graham Jessica, Baldwin Lara, Harrigan Suzy, Hok Pan Yuen, Fornito Alex, Allott Kelly, Alvarez-Jimenez Mario, O’Donoghoe Brian

**Affiliations:** 1 Orygen Youth Health Research Centre for Youth; 2 Monash University

## Abstract

**Background:**

While antipsychotic medication (AP) is a very effective treatment for positive psychotic symptoms in first-episode psychosis (FEP), it is also associated with risks. These include adverse neurological and metabolic effects and measurable changes in brain structure. APs may even be associated with poorer functional recovery. Due to advances in the detection of, and psychosocial treatments for FEP, it is now ethically feasible to study the relative risks and benefits of offering AP as a first line treatment, and conversely, of withholding it, on a background of comprehensive evidence-based psychosocial care. This non-inferiority design randomised double blind placebo controlled study examines whether a (low-risk) subgroup of people with FEP can recover without AP, and considers the effects on functioning, physical health, cognition, and brain structure of AP versus withholding AP.

**Methods:**

Young people with FEP were screened for study eligibility and invited to participate if they met stringent inclusion criteria indicating low-risk of harm to self or others, and adequate social support. Hence a large proportion of patients were assumed a priori to be too high risk to withhold antipsychotic medication. Participants were randomly assigned to receive either low dose AP (MIPT group) or placebo (PIPT group) for six months, and all participants received intensive psychosocial treatment. Randomisation was stratified with three levels of DUP and gender creating six cells. Assessments of psychopathology, neurocognitive performance, and neuroimaging occurred regularly until two years after study entry.

**Results:**

90 young people were randomised and 81 commenced trial medication. They were 44% male and mean age 18.5 years (SD = 2.7). Thirty-four percent of participants completed the six month medication phase and there were more completers in the placebo group than the medication group. On the primary outcome measure of SOFAS there was significant evidence that the placebo group was not inferior to the medication group (SOFAS: MIPT mean = 61.5, SD = 13.4; PIPT mean = 61.7, SD = 16.8). The two groups were found to be very similar on all psychopathology assessments and measures of functioning at both baseline and following treatment, suggesting that the outcomes of the two treatment regimes were not different with respect to symptoms and functioning.

**Discussion:**

The results of this study demonstrate that it is feasible and acceptable to conduct AP-free research in carefully selected FEP to examine the risk-benefit ratio of current treatments under carefully controlled conditions that prioritise patient outcomes and safety. Although only one-third of the participants completed the six month trial intervention period, more of those on placebo completed the trial phase and they had higher mean, minimum and maximum time in the experimental intervention phase than those on medication. In addition, there were no differences between the groups on measures of psychopathology and functioning, suggesting that the intensive psychosocial intervention provided to all participants is complementary and may be more important than antipsychotic medication in the early phases of psychotic illness for a subgroup of young people. However this subgroup is very small as a % of total FEP patients treated during the study period. Further analysis of physical health and neuroimaging data and completion of the 24 month follow-up assessments will allow detailed examination of the risk-benefit ratio regarding antipsychotic medication in FEP.

